# Are We Ready to Implement Molecular Subtyping of Bladder Cancer in Clinical Practice? Part 1: General Issues and Marker Expression

**DOI:** 10.3390/ijms23147819

**Published:** 2022-07-15

**Authors:** Francesca Sanguedolce, Magda Zanelli, Andrea Palicelli, Stefano Ascani, Maurizio Zizzo, Giorgia Cocco, Lars Björnebo, Anna Lantz, Ugo Giovanni Falagario, Luigi Cormio, Giuseppe Carrieri

**Affiliations:** 1 Pathology Unit, Policlinico Riuniti, University of Foggia, 71122 Foggia, Italy; 2 Pathology Unit, Azienda USL-IRCCS di Reggio Emilia, 42123 Reggio Emilia, Italy; magda.zanelli@ausl.re.it (M.Z.); andrea.palicelli@ausl.re.it (A.P.); 3 Pathology Unit, Azienda Ospedaliera Santa Maria di Terni, University of Perugia, 05100 Terni, Italy; s.ascani@aospterni.it; 4 Surgical Oncology Unit, Azienda USL-IRCCS di Reggio Emilia, 42123 Reggio Emilia, Italy; maurizio.zizzo@ausl.re.it; 5 Radiotherapy Unit, Policlinico Riuniti, 71122 Foggia, Italy; gcocco@ospedaliriunitifoggia.it; 6 Department of Medical Epidemiology and Biostatistics, Karolinska Institutet, 171 77 Solna, Sweden; lars.bjornebo@ki.se (L.B.); anna.lantz.1@ki.se (A.L.); 7 Department of Molecular Medicine and Surgery, Karolinska Institutet, 171 77 Solna, Sweden; 8 Department of Urology and Renal Transplantation, Policlinico Riuniti, University of Foggia, 71122 Foggia, Italy; ugofalagario@gmail.com (U.G.F.); luigi.cormio@unifg.it (L.C.); giuseppe.carrieri@unifg.it (G.C.); 9 Department of Urology, Bonomo Teaching Hospital, 76123 Andria, Italy

**Keywords:** bladder cancer, molecular classification, immunohistochemistry

## Abstract

Bladder cancer (BC) is a heterogeneous disease with highly variable clinical and pathological features, and resulting in different outcomes. Such heterogeneity ensues from distinct pathogenetic mechanisms and may consistently affect treatment responses in single patients. Thus, over the last few years, several groups have developed molecular classification schemes for BC, mainly based on their mRNA expression profiles. A “consensus” classification has recently been proposed to combine the published systems, agreeing on a six-cluster scheme with distinct prognostic and predictive features. In order to implement molecular subtyping as a risk-stratification tool in routine practice, immunohistochemistry (IHC) has been explored as a readily accessible, relatively inexpensive, standardized surrogate method, achieving promising results in different clinical settings. The first part of this review deals with the steps resulting in the development of a molecular subtyping of BC, its prognostic and predictive implications, and the main features of immunohistochemical markers used as surrogates to stratify BC into pre-defined molecular clusters.

## 1. Introduction

### 1.1. Clinical Aspects of Bladder Cancer

Bladder cancer (BC) ranks seventh among the most prevalent tumors worldwide, in both sexes and all ages, with an estimated number of prevalent cases as high as 1,720,625, and it stands among the 10 leading causes of cancer death in the old adult population (≥60 years) [1].

Muscle-invasive bladder cancers (MIBCs, namely T2-T4 disease) account for approximately a quarter of all BCs, nonetheless they carry the highest mortality rates [2,3]. Therefore, radical cystectomy (RC) with pelvic nodal dissection and neoadjuvant or adjuvant chemotherapy are used in the therapy of MIBC [2], whereas immune checkpoint inhibitors and targeted therapies have been recently introduced as novel treatment options in a subset of patients [4,5]. A high rate of non-responders has been reported in patients undergoing neoadjuvant chemotherapy (NAC) [6,7], with inherent imbalance between inconsistent clinical benefit and considerable side effects, along with a delayed subsequent treatment in these cases [8,9], and the currently available data do not support the use of immunotherapy in the neoadjuvant setting [2].

Non-muscle-invasive bladder cancer (NMIBC), encompassing Ta, T1, and urothelial carcinoma in situ (CIS), accounts for 75–80% of all BCs [10]. This is a heterogeneous group of tumors, including both low- and high-grade non-invasive BCs, along with superficially invasive (stage pT1) tumors, with variable rates of recurrence and progression to higher-stage disease, the latter occurring in 31–78% of all NMIBC patients [10,11]. The main treatment options for NMIBCs include transurethral resection, intravesical instillations, and/or patients’ follow-up with cytology and repeated cystoscopies [10,12]. Intravesical administration of bacillus Calmette–Guérin (BCG) is the mainstay of treatment in high-grade NMIBC, though treatment failure occurs in a substantial number of cases [13], due to the development of resistance over time, or to toxicity [14,15], thus warranting a more aggressive treatment with RC [10].

Risk stratification is currently performed using scoring models based on clinical and pathological parameters [10,16] which inform patients’ treatments and surveillance plans [10,11,16]. Nevertheless, differences resulting from biological features and molecular subtyping, which are the main causes of such clinical variability, cannot be assessed by available risk-stratification systems. Attempts have been made to assess the prognostic role of single to combined markers, both in MIBCs and in NMIBCs, with controversial results [17,18,19], including a recent multicenter retrospective analysis on the impact of pentafecta on oncological outcomes of patients undergoing RC [20]. Since all available treatment options carry side effects and/or risk of failure, the need for reliable biomarkers for patients’ risk stratification and therapeutic management to be applied in clinical practice is still unfulfilled.

### 1.2. Molecular Subtyping of Bladder Cancer

Over the last decade, several attempts have been made by different groups in order to stratify BC into molecular subtypes using mRNA expression profiling, mirroring major intrinsic subtypes defined in breast cancer [21,22,23,24,25,26,27,28], with the majority of studies focusing on MIBC (Table 1).

A first approach to BC molecular classification was made by the MD Anderson Cancer Center group; they investigated the whole-genome mRNA expressions of a cohort of muscle-invasive (MI) BCs, describing the two basal and luminal subtypes [23], which were named after the gene-expression signature of normal basal and luminal urothelial cells. The basal subgroup (*CK5*/*CK6*+/*CK14*+/*P63*+) is enriched with squamous features, presents at advanced stage, and carries a worse prognosis, yet it is highly sensitive to cisplatin-based chemotherapy. Luminal BCs usually features papillary morphology, carries *FGFR3* mutations, and activation of the peroxisome proliferator activator receptor γ (*PPARγ*) pathway, and is less aggressive than the former, though it may show resistance to chemotherapy in some cases.

Later, the Cancer Genome Atlas (TCGA) group comprehensively analyzed a series of MIBCs by multiple molecular platforms, resulting in the recognition of five molecular subtypes, namely, luminal–papillary, luminal–infiltrated, luminal, basal–squamous, and neuronal subtypes [24], with inherent clinical implications; due to their peculiar gene-expression profiles, the luminal–papillary subtype is poorly sensitive to cisplatin-based chemotherapy, yet treatment with *FGFR3* tyrosine kinase inhibitors might be effective in this setting, whereas the luminal-infiltrated subtype might respond favorably to immune checkpoint therapy. The basal–squamous subtype is likely sensitive to both cisplatin-based chemotherapy and immune checkpoint therapy. The neuronal subtype is distinguished by robust expressions of neuroendocrine and neuronal genes and frequent mutations in cell-cycle genes *TP53* and *RB1*, although it does not show the conventional neuroendocrine morphology. This subtype exhibits the worst survival rates, nevertheless it may respond to etoposide–cisplatin chemotherapy.

A series of widely overlapping molecular classification systems has been proposed over the years, each proposing a distinct nomenclature [22,23,24,29,30,31,32].

Despite inherent differences in subtypes nomenclature and number, resulting from the use of disparate methodologies and interpretation criteria, the top-level distinction into luminal and basal subtypes is common to all classification systems, and results from the identification of molecular features of normal basal stem cells and luminal (intermediate and superficial) cells, respectively, in such tumors. These two major molecular phenotypes echo the presence of underlying different oncogenic pathways [33,34,35], as conformed by studies on murine models of bladder carcinogenesis [36,37,38], since luminal and basal invasive BCs mostly develop either via the papillary pathway, probably through non-invasive papillary lesions, or via the nonpapillary track, respectively [39].

These subtypes are enriched for peculiar clinical and pathological features, thus may provide prognostic and predictive information to better refine risk stratification [21,22,23,24,40,41]. Whereas the basal type remained mostly stable over the different classifications, the luminal subtype may be further split into urothelial-like (URO) and genomically unstable (GU) [21,27,41,42,43], as well as into smaller subclasses, from time to time.

Recently, a consensus classification resulting from an international joint effort has been developed, in order to provide a common framework for the molecular subtyping of MIBC [25]; through the meta-analysis of 1750 MIBC transcriptomic profiles from 18 published datasets, six molecular clusters have been identified, namely, luminal–papillary (LumP, accounting for 24% of all MIBCs), luminal non-specified (LumNS, 8%), luminal-unstable (LumU, 15%,) stroma-rich (15%), basal–squamous (Ba/Sq, 35%), and neuroendocrine-like (NE-like, 3%). These subtypes have distinct clinical outcomes, pathological features, immune microenvironments, and patterns of gene expression, resulting in different potential responsiveness to treatment (Table 2). There is a striking variability in median overall survival across these groups, ranging from 4, 3.8, and 2.9 years (LumP, stroma-rich, and LumU, respectively), to 1.8, 1.2, and 1 years (LumNS, Ba/Sq, and NE-like, respectively) [25]. LumU, Ba/Sq, and NE-like tumors were enriched for *P53* mutations, whereas *FGFR3* mutations were most frequent in the LumP subtype.

Since tumors belonging to distinct subtypes may be differentially responsive to oncological treatments, several studies focused on the predictive role of molecular subtyping [24,25]. Two recent studies failed to show any prognostic impact of molecular subtyping, either gene expression-based or immunohistochemistry-based, in terms of survival, in BC patients who underwent RC [44,45]. According to Weyerer et al. [45], a putative reason may be the lack of homogeneity in the previous studies, in that they included both NMIBCs and MIBCs, in the absence of a systematic pathological revision. Interestingly, in the few studies assessing the role of molecular subtyping in predicting response to neoadjuvant chemotherapy, no significant differences were reported among distinct groups in terms of survival, possibly due to the clinical benefit from NAC [40,46,47].

Conversely, results from the phase II clinical trials of IMvigor210 and CheckMate 275 demonstrated different response rates in patients with advanced urothelial carcinoma (UC) treated with immune checkpoint inhibitors atezolizumab and nivolumab, respectively, according to molecular subtyping [48,49,50,51].

## 2. Implementing Molecular Subtyping of Bladder Cancer in Clinical Practice

### 2.1. General Features

Whole-transcriptome subtyping may provide useful prognostic and predictive information, nevertheless, such tests are complex, time- and money-consuming, and not easily accessible, thus their clinical implementation is not currently feasible [3,25]. An immunohistochemical algorithm ready to be applied to the pathology workflow would be a powerful tool for assessing a patient’s prognosis, improving risk stratification in NMIBCs, and reducing the economic burden and discomfort of repeated cystoscopies. Based on the available data from the literature, which will be discussed in the following sections and sub-sections, in our own practice we have recently introduced a four-panel antibody encompassing CK20 and GATA3 as luminal markers, and CK5/6 and CK14 as basal markers. 

Since immunohistochemistry (IHC) is now a universally acknowledged, standardized ancillary method, IHC-based subtyping has the main advantage to be performed in the vast majority of pathology laboratories. The antibodies used in the recently proposed algorithms as surrogates to molecular markers are readily accessible and relatively inexpensive and have been implemented in routine diagnostics [52,53,54]. 

Furthermore, IHC has the advantage to visualize the location of single markers, thus allowing to distinguish between signals from non-tumor cells (i.e., stromal cells, immune cells), and those from cancer cells, which may severely affect the reliability of molecular subtyping based on gene-expression profiling, especially in the setting of highly infiltrated tumors [31,40,42,43,55].

Another major edge of IHC-based profiling is that it does not require fresh tissue, which may be difficult to retrieve in the clinical setting, but it is routinely performed in formalin-fixed paraffin-embedded (FFPE) specimens, thus it can be applied to archival material [56].

When comparing protein- and RNA-base methods to perform subtype classification, divergence and convergence phenomena should be taken into account [27,55], in that tumors belonging to the same IHC-based subtype may cluster apart by mRNA analysis (divergence), whereas tumors with different phenotypes may co-cluster at RNA level (convergence), possibly due to the presence of low or high amounts of signals from non-tumor cells, and/or the gain of overlapping molecular features with increasing stage. Such issues contribute to the conflicting results reported so far in comparing subtyping assessed at mRNA and protein levels in MIBCs, thus prompting the suggestion that a bi-nominal classification model encompassing both tumor cell phenotype and gene-expression clusters would be more effective in stratifying these tumors [27].

With the increasing application of artificial intelligence (AI) in surgical pathology, IHC scoring may be performed digitally as well, thus improving the reproducibility of the interpretation of staining results [57].

The effectiveness of IHC in aiding the molecular classification of BC needs to be carefully assessed in view of (1) the actual clinical impact of the differences between gene expression-based and IHC-based subtyping, (2) the assessment of staining on tissue microarray (TMA) rather than whole slides, thus leading to possible false-negative results because of tumor heterogeneity [58], and (3) the accurate selection of immunohistochemical markers which may increase the reliability of this method. This will be obtained through validation of selected antibody panels, with proper cut-offs, on large, independent cohorts [59], in order to consistently and reliably stratify these tumors.

### 2.2. Using Immunohistochemistry-Based Models to Subtype Bladder Cancer

On the basis of their gene signatures, GATA3 and CK20, and CK5 or CK5/6 and CK14 are conventionally used as markers of the luminal and basal subtypes, respectively [23,28,33,60,61], and P16 may be added to further stratify luminal subtypes into URO and GU [27,57,62], mostly yielding significant association with survival outcomes in keeping with their molecular counterparts [63]. In MIBC, the GU subtype has been reported to be associated with poor prognosis along with a higher mutational burden and greater immune infiltration, resulting in increased responses to immune checkpoint inhibitors as second-line treatment [64], according to a phase 2 clinical trial (IMvigor210).

An attempt was made to develop an IHC-based subtyping model by Lund University group, consisting of a large antibody panel (28), with the aim to define 10 subgroups mirroring the luminal (including URO and GU), basal, mesenchymal-like, and neuroendocrine (NE)-like categories [21,27]. Nonetheless, to be implemented in clinical practice, an optimal antibody panel should include a limited number of IHC markers.

#### 2.2.1. Immunohistochemistry-Based Subtyping of MIBC

In a previous study, Sjodahl et al. reported on the utility of two immunohistochemical markers (CK5, CCNB1), pathologic grade, and urothelial-like growth pattern carcinoma, to stratify tumors into three major subtypes (Uro, encompassing UroA and UroB, GU, and squamous cell cancer-like, or SCCL), formerly suggested by whole-genome gene-expression analysis. Their results were obtained upon unsupervised selection of classifier variables, including 20 immunohistochemical markers and clinicopathologic data from 237 BCs. The IHC-based subtyping was consistent with results obtained by gene-expression profiling, as confirmed by survival analyses [65].

Rebouissou et al. developed a 40 gene-based transcriptomic signature and selected a two-antibody panel, including CK5/6 and FOXA1, with a sensitivity and specificity as high as 89% and 95.5%, respectively, in their series of MIBCs, which was further validated in an independent cohort [30].

Choi et al. demonstrated a high concordance between the array-based measurements of basal- and luminal-marker expression and the results obtained with quantitative RT-PCR and IHC in their study cohort. Furthermore, their TMA analysis of 332 pT3 BCs showed that the coordinated expression of CK5/6 and CK20 might reliably stratify tumors within the basal and luminal subgroup [23]. Using approximately the same two-antibody panel (CK5 and CK20), Sikic et al. identified four subgroups out of their cohort of 222 upper-tract urinary cancers (UTUCs), namely, luminal (CK5-/CK20+), basal (CK5-/CK20+), double-negative (DN), and double-positive (DP), the latter two accounting for the majority of cases (54.9%) [66]. Interestingly, they reported a significantly worse cancer-specific survival (CSS) in the luminal subgroup as compared to the others, in keeping with the results from the comprehensive transcriptional analysis by Hedegaard et al. [26].

In their large meta-analysis, Dadhania et al. performed supervised hierarchical clustering of basal and luminal biomarkers using whole transcriptome expression data from a large series of 937 BC samples of different stages and analyzed the consistency of a series of immunohistochemical markers in classifying tumors into luminal and basal molecular subtypes. Finally, they identified CK5/6-GATA3 and CK14-GATA3 as the two pairs of markers yielding the highest accuracy (91% and 89%, respectively) [33].

Accordingly, Hodgson et al. stained a cohort of 207 cases of high-grade BCs for CK5/6 and GATA3, resulting in 85.2% and 14.8% luminal (CK5/6-/GATA3+) and basal (CK5/6+/GATA3-) tumors, respectively, the latter being associated with worse disease-specific survival (DSS,) as well as a higher amount of CD8+ lymphocytes, and expression of PD-1 and PD-L1 [67]. Recently, Guo et al. [28] recommended the same two-antibody panel, including GATA3 and CK5/6, developed out of five candidate markers, as a tool to effectively classify luminal and basal molecular subtypes with up to 92% reliability as compared to transcriptomic analysis. In keeping with that, Bejrananda et al. classified their cohort of MIBCs into luminal, basal, and DN cases by the same two-antibody panel with 62% accuracy, and the addition of two further luminal (CK20) and basal (CK14) markers did not improve survival prediction [68]. Using this simple classifier with the addition of P16, Olkhov-Mitsel et al. [56] recently assessed a cohort of 243 MIBC patients treated with RC alone, classifying almost all of them (97.1%) into three subtypes, namely, Uro (GATA3+/CK5/6-/P16-), GU (GATA3+/CK5/6-/P16+), and Basal (GATA3-/CK5/6+), which should correspond to the LumP and LumNS (Uro), LumU (GU), stroma-rich, and Ba/Sq (Basal) groups, according to the recent consensus classification [25]. In the study by Serag Eldien et al., subtyping based on a GATA3/CK5/6 antibody panel revealed no significant impact on overall survival (OS) and progression-free survival (PFS), although a trend to better survival for luminal tumors compared to other groups was noticed [69], in keeping with other studies [70]. Variation in staining techniques and interpretation may partly explain this disparity in results [68].

#### 2.2.2. Immunohistochemistry-Based Subtyping of NMIBC

Through comprehensive transcriptional analysis, basal NMIBCs, enriched for higher RNA expression of *CK5*, *CK6*, and *CD44*, were reportedly associated with PFS in comparison with their luminal counterparts, which, in turn, showed increased RNA expression of *CK20*, *FOXA1*, and *GATA3* [26]. Accordingly, a subsequent study on pT1 NMIBC described a higher proliferative activity and worse recurrence-free survival (RFS) and PFS in a subset of luminal tumors with *CK20*high/*CK5*low RNA expression [71], in keeping with other studies [26,72,73,74]. Furthermore, the cohort of pure high-grade NMIBCs assessed by Schnitzler et al. featured a luminal-like phenotype, along with a low *FGFR3*/*CDKN2A* alteration frequency and a high rate of mutations in genes encoding chromatin-modifying proteins [75]. It has been suggested that such clinically aggressive luminal-like NMIBCs might be enriched for aberrations in junctional complexes, high level of copy number alteration [26,76,77], along with the cell cycle, proliferation, and progression gene sets [78].

Jackson et al. described a simple three-antibody immunohistochemical algorithm, including GATA3, CK5, and P16, to classify NMIBC into four distinct subtypes [79], identified through unsupervised hierarchical clustering analysis, namely, basal, URO, GU, and URO-KRT5+ [27,57,62,80]. Of them, the URO-KRT5+ subtype, which accounted for 23% cases in their series, was a novel one, enriched for low-stage, low-grade, slow-recurring tumors, and characterized by a GATA3+/suprabasal CK5+ immunohistochemical profile, in keeping with class 3 NMIBCs described by Lindskrog et al. in a recent series [81]. Conversely, CK5+/GATA3- basal tumors and CK5-/GATA3+/P16- URO tumors showed lower PFS and RFS, respectively, compared to the other subtypes. In keeping with this, Lu et al. recently described a significant association with worse RFS and PFS of a subset of CK20 low/GATA3 low basal/squamous tumors within their cohort of pT1NMIBCs [82]. Patschan et al. examined 149 T1 NMIBCs using the model developed by Sjodahl et al. [65] (see previous section) to stratify the whole cohort into Uro, GU, and SCCL subtypes, with prevalence rates as high as 32%, 58%, and 10%, respectively [41]. Additional markers were performed, revealing that Uro tumors were enriched with FGFR3, CCND1, P63, and RB1 protein expression, and GU tumors with HER2, KI67, P16, and E2F3 markers. According to their analysis, GU and SCCL NMIBCs had a high CD3+ lymphocyte infiltration, and high EORTC scores, as well as a strong tendency to progress, in keeping with the significant association between progression biomarkers assessed at the mRNA level and molecular subtypes. Since IHC allows to identify the staining location, CK5 expression to basal cell layers was present in a low-risk subset of tumors, consistent with the findings by Jung et al. [83].

In their recent study, Muilwijk et al. assessed a selected cohort of pTa NMIBCs by an antibody panel, including CK5, P63, P40, and GATA3, and CK20 as basal and luminal markers, respectively. Interestingly, GATA3 was expressed in all specimens, whereas CK5 and CK20 expressions showed a significant inverse correlation; furthermore, CK5 staining was consistent with its RNA expression. Such CK5/CK20 subtyping model was reportedly effective in discriminating between low-grade and high-grade NMIBC, since the latter had a significant inverse correlation with CK5 expression and positive correlation with CK20 expression, yet failed in predicting disease outcome, possibly due to the low number of events in their cohort [84]. Accordingly, a recent study applying an IHC-based classifier for luminal (GATA3, CK20, ER, Uroplakin II, and HER2) and basal (CK5/6 and CD44) markers did not identify any prognostic role in NMIBC [85].

Further studies on non-muscle-invasive UTUC found that CK5 negativity is an independent prognostic factor in this setting [66,83,86]. Jung et al. reported that lack of CK5/6 expression was a strong independent prognostic factor of shorter PFS and CSS in a cohort of non-muscle-invasive UTUCs. Furthermore, luminal-like subtypes (CK5/6-/CK20+ and CD44-/CK20+) were associated with poor outcomes [83]. Accordingly, Mai et al. identified a subset of basal NMIBCs upon their reactivity for CK5 and CD44, which accounted for 12.9% and 17.4% of low- and high-grade tumors, respectively. These basal NMIBCs had significantly increased rates of multifocality, recurrence, and progression to higher grade and stage, as compared to the non-basal subgroup [87].

Such conflicting data regarding the prognostic role of basal and luminal antibodies, especially CK5, in NMIBCs, led to the hypothesis that this marker may be either positively or inversely correlated to high-risk biology in different NMIBC subsets, according to its pathway of expression [41,84].

Few attempts to subtype selected cohorts of CIS lesions were made, and an overall lack to very low expression of basal markers (CK5/6, CK14) was reported [88,89], along with a significant degree of intratumor heterogeneity. Furthermore, no correlation with clinical outcome was identified [89]. This is in keeping with the diagnostic role of a strong and diffuse CK20 staining in distinguishing CIS from other flat urothelial lesions [90].

Interestingly, a recent study reported on a significantly increased PFS in a subset of basal/squamous pT1 NMIBC patients treated with gemcitabine plus cisplatin intra-arterial chemotherapy as compared to patients with luminal A and B tumors (*p* = 0.263 and *p* = 0.313, respectively), and to those who received intravesical chemotherapy (*p* = 0.011) [82], possibly due to the higher expression of the lnRNA NEAT1 and significant induction of the transcription factor EGR1 described in basal-like NMIBCs by comprehensive transcriptional analysis [26], since both molecules are involved in the chemosensitivity mechanisms of other malignancies, such as lung, ovarian, and esophageal cancers. 

Overall, the apparently controversial findings concerning the role of molecular subtyping in NMIBCs may be due to several reasons, namely, (1) the use of one or more different methods, such as immunohistochemistry and/or RNA assessment by RT-qPCR [71], (2) the variability in tumor stage and grade across cohorts, namely, CIS/Ta/T1 and low-grade/high-grade [72], and (3) the assessment of intratumoral heterogeneity [91,92], the latter being affected by the technique used, such as TMA vs. whole-slide analysis.

### 2.3. Heterogeneity in Bladder Cancer

Heterogeneity is definitely a feature of BC, first at the clinical level, due to the high variability in its clinical presentation [3], as well as in its histological characteristics, as shown by the presence of a spectrum of morphological variants [93]. Molecular approaches support such phenotypical heterogeneity, demonstrating a wide spectrum of DNA copy number changes, genomic mutations, and alterations in methylation profiles [24,94], as demonstrated through the analysis of multifocal tumors within the same bladder [91].

The occurrence of variant histology within the same tumor poses the issue of intratumor heterogeneity, as supported by variability in phenotypical and molecular features [95,96]. Intratumor heterogeneity has been reported by Warrick et al. [92], who described distinct IHC-based subtypes in different areas from the same tumor presenting conventional urothelial carcinoma (UC) and histologic variants, with frequent co-occurrence of Ba/Sq tumors with either Uro or GU UCs [92]. Although the current consensus classification addresses only the major molecular subtype, assessing heterogeneity in a tumor may be important since it might result in conventional chemotherapy resistance and in targeted therapies failure [25]. 

Furthermore, such differences may be seen by comparing primary tumors and their matched metastases, mostly in the Ba/Sq subtype, though at low levels [70,97,98,99], possibly due to (1) the prevalence of single clones within heterogeneous primaries, (2) protein expression switches due to chemotherapy-induced enrichment in mutations, and/or (3) interactions with the microenvironment in the two different anatomical sites [70,99,100]. In keeping with that, high concordance rates were described in a recent study assessing primary chemotherapy-naïve BCs and their metastases by the expression of luminal (FOXA1, GATA3) and basal (CK5/6, CK14) markers [101].

Intratumor heterogeneity at the molecular level is difficult to assess, nevertheless, it should be taken into account in patients’ management, since it may result in treatment failure [98].

Intratumor heterogeneity has been recently assessed in Ba/Sq tumors using a combined genomic and immunohistochemical approach, with the latter method yielding good results in identifying underlying changes in mRNA expression [98], in keeping with previous reports [56]. In the recent study by Sirab et al. [98], a NanoString panel for a limited number of genes and a two-antibody immunohistochemical panel, including KRT5/6 and GATA3, was used to identify Ba/Sq tumors, yielding consistent results, thus pointing out the validity of IHC in the assessment of heterogeneity in this setting [98]. Their results showing the divergent expression of genes underlying luminal/basal differentiation among immunohistochemical heterogeneous area in the same tumor suggest the presence of hierarchical states of differentiation within the tumor, as well as a pathogenetic mechanism of clonal evolution [98,102,103]. In this setting, whole-slide imaging and software analysis might implement IHC-based tumor profiling.

All in all, the prognostic and predictive role of detecting intratumor heterogeneity, especially in Ba/Sq MIBC, should be further assessed by prospective analysis and clinical trials of patients amenable of neo-adjuvant chemotherapy or immune checkpoint therapy. Moreover, potential differences between tissue-based and circulating tumor markers should be taken into account, especially in view of analyzing liquid biopsies instead of, or along with, tissue specimens [104].

Heterogeneity was examined in studies focusing on NMIBC tumors, yielding interesting results. Using immunohistochemical antibodies as surrogate markers for molecular subtyping, Barth et al. described a luminal-to-basal-like switch in a series of CIS lesions developing invasion of the lamina propria [88]. Accordingly, intertumoral heterogeneity among CIS lesions from the same patient has been reported by Garczyk et al. [89]. A practical issue would be the evaluation of tumor foci with different immunophenotypical profile within the same patient; a conceivable solution would be either assessing the lesion with the highest stage and grade or taking into consideration the worst subtype only. However, further focused studies are needed to specifically address this point.

**Figure 1:**
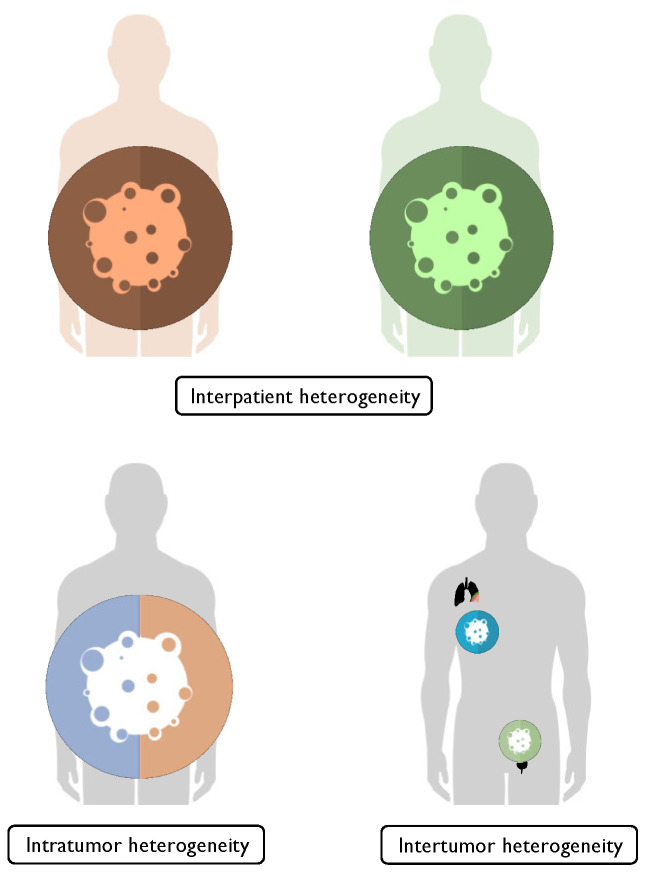
Different types of bladder cancer heterogeneity (see text).

Interestingly, two recent studies on NMIBCs [80] highlighted the stability of subtypes over time (temporal homogeneity) and among different cores from the same patients (intertumoral homogeneity), arguing against tumor heterogeneity as a limitation in immunohistochemical classification [79] (Figure 1).

## 3. Combining Immunohistochemical Markers to Subtype Bladder Cancer

### 3.1. Expression of Markers in Non-Neoplastic and Neoplastic Urothelium

Normal urothelium has a bottom-up (pseudo)stratified structure encompassing basal, intermediate, and umbrella cells with increasing differentiation implying changes in keratin profiles (see below); accordingly, UCs developing from such distinct cell types have different expression signatures and immunophenotypes [33,35,105], providing ground for the development of the molecular subtyping of BC. 

Specifically, CK20, GATA3, and UPK3 expression is restricted to the more differentiated cells in normal urothelium, whereas intermediate- to high-molecular-weight cytokeratins 14 and 5/6 are present in basal urothelial layers adjacent to the basement membrane; intermediate cells exhibit variable staining for CK5/6, and are usually CK14-/CK20- [33,35,65]. The basal layer encompasses pluripotent stem cells or uroprogenitor cells important for normal functions of homeostasis and orderly regeneration after injury, whereas intermediate to terminally differentiated cells gradually lose their proliferating potential according to a precise and constant differentiation program, resulting in arrest of CK5 expression and onset of CK20 expression [39,106]. It has been suggested the longer lifespan of basal cells results in the potential occurrence of multiple genomic alterations [22].

The vast majority of BCs fall into the basal/luminal dichotomy, with inherent prognostic and predictive implications. Overall, molecular and immunophenotypical features of luminal and basal tumors suggest their developing via the papillary and non-papillary pathway, respectively [30]. Different antibodies have been used to identify tumors belonging to different molecular classes, mostly to the luminal and basal subgroups (Table 3).

On the basis of a literature search, we identified a series of immunohistochemical markers, which we are going to briefly discuss in the following sub-sections.

### 3.2. CCND1

*CCND1*, encoding for cyclin D1, is a member of the cyclin family of cell-cycle regulators. It has been included in an early antibody panel proposed by Lund University to subtype BCs [21,27] in a series of subgroups broadly correlating with molecular-based categories. In this setting, CCND1 expression discriminates between Uro (CCND1+/FGFR3+/RB1+/p16-) and GU (CCND1-/FGFR3-/p16+/RB1-) tumors [43,60,109]. CCND1 antibody has been reported to stain the nuclei of cells located at the basal and intermediate cell layers up to all tumor cells in urothelial-like tumors [109], while being absent or low-expressed in the vast majority of GU and SCCL tumors [65]. Such differential expression of selected markers results from the variable activity of different genes in the early (*CCND1*, *RB1*) and late (*p16*) phases of the cell cycle in Uro and GU tumors, respectively [21,27,31].

Among NMIBCs, the expression of CCND1 as well as other cell-cycle-related markers has been significantly associated with better clinical outcomes in terms of PFS, and inversely related to stage and grade, in keeping with its association to the UroA subtype [26,31,55], as confirmed by complex expression signatures and IHC [110,111,112]. Consistently, a mutational rate as high as 72% has been reported in CIS specimens, involving genes of the *TP53*/cell cycle pathway, mainly from genomic alterations in *CCND1* [113].

Higher CCND1 expression was significantly associated with poor prognosis in two cohorts of MIBCs [55,114]. Interestingly, in a study assessing the heterogeneity in IHC-based subtyping between primary BCs and matched nodal metastases, the strongest positive correlations were seen for CCND1 and RB1 [97]. Conversely, Dadhania et al. described overlapping expression of a series of immunohistochemical markers, including CCND1, to the extent that they were not chosen to discriminate between luminal and basal tumors [33]. In another study, basal-like BCs were significantly enriched with *CCND1* amplification [22].

### 3.3. CK5/6

CK5/6 (encompassing both CK5 and CK6) is a marker of stratified squamous epithelia and, within the urothelium, it stains basal and intermediate cells. Overall, CK5/6 immunoreactivity in conventional BC is highly variable (approximately 20–60%) [54,69,115,116], and significantly higher in squamous tumors [69,117]. CK5 staining is often more intense and readily detectable as compared to other basal markers, such as CD44 [87].

In previous studies from the Lund group, CK5 expression was identified in as many as 91% UroA tumors, with significantly higher rates in UroA than in UroB tumors (49% versus 10%, *p* < 0.002) [65]. Staining for CK5 was restricted to the basal cell layer in Uro tumors, both in the MIBC and NMIBC setting [41,65]. Conversely, in SCCL tumors a strong, diffuse CK5 expression was detected in 92% of cases, whereas most GU tumors lacked CK5 expression [65].

CK5/6 expression has been reported to be significantly associated with muscle and perineural invasion [69,118], and with poorer survival in several reports [115,118], whereas the opposite has been described in studies on UTUC [118,119]. Bejrananda et al. reported on significantly worse survival outcome in patients with loss of CK5/6 expression [68]. Conversely, increased CK5 expression was significantly associated with old age, muscle invasion, and stromal inflammation in a cohort of 90 BCs [120]. Accordingly, in the study by Mai et al. [121], the cohort of CK5-positive tumors, labeled as “basal”, showed higher rates of nodal and distant metastases as compared to CK5-negative BCs, though a statistical analysis was not provided.

### 3.4. CK14

CK14 is a type I acidic keratin that is expressed in mitotically active basal cells of the stratified epithelium, where it promotes proliferation and differentiation, and supports structural integrity [122]. As CK14-positive basal cells differentiate into umbrella cells in the normal urothelium, CK14 expression is down-regulated and replaced by CK20. Consistent with this observation, Volkmer et al. [35] and Ho et al. [105] demonstrated that CK14 defined the most primitive/least differentiated basal-type urothelial carcinoma, which preceded the emergence of cancer cells expressing CK5 (intermediately differentiated) or CK20 (well-differentiated); further, CK14 expression marked the highly tumorigenic stem cell population. The increase in CK14 immunoreactivity was also observed at an early carcinogenesis stage, initiating the appearance of malignant lesions of the urinary bladder in a rat model [123]. Thus, the use of CK14 as a marker alternate to CK5/6 in identifying basal-like tumors at the protein level could not be effective [28], since its expression would be rather useful in identifying early BCs carrying a stem cell signature and inherent aggressive features [35,124]. According to the Lund classification, CK14 expression was significantly higher in UroB tumors as compared to UroA tumors (30% versus 9%, *p* < 0.01), with positive staining restricted to basal cells [65]. The highest expression rate was seen in the SCCL subtype (76%), with a moderate-to-strong, diffuse staining, whereas GU tumors showed absent to patchy CK14 staining. Overall, CK14 expression has been described in approximately half of UCs [120]. In one study CK14 expression was significantly associated with PNI and muscle invasion [120].

Jung et al. assessed CK14 expression in their cohort of 204 papillary NMIBCs, reporting a significant association with high-grade, advanced stage, high proliferative index, poor PFS, squamous cell cancer (SCC) development, along with *TNF-α*, *NF-kB,* and *P53* pathways [124]. Accordingly, high CK14 levels were independently prognostic of poor survival both in NMIBC and in MIBC [35], in keeping with reports from studies focused on other malignancies, including breast cancer, squamous cell carcinoma, and salivary gland carcinoma [124].

### 3.5. CK20

Traditionally, UC has been regarded as a tumor showing combined staining for both CK7 and CK20; nonetheless, overall rates of CK20 expression are highly variable across studies, ranging from 24.4% in tumors arising in the upper urinary tract to approximately 70% in cohorts of BC [66,120,125,126,127].

CK20 is a marker of urothelial differentiation, which has been suggested to be aberrantly expressed within the tumor parenchyma according to its distance from the stromal compartment [34]. However, CK20 expression was abnormally located to the intermediate urothelial layers in 72%, 56%, and 47% of GU, UroA, and UroB tumors, respectively, according to the Lund classification [65]. Only 3 SCCL tumor stained positive for CK20.

CK20 has been reported to be correlated with higher tumor grade and stage in papillary UC [128]. The presence of an absent to aberrant staining for CK20 in their subset of advanced urothelial-like BCs, defined on the basis of a urothelial differentiation mRNA signature, led Sjodahl et al. to hypothesize a corruption of the normal differentiation program over tumor progression, which the authors define as “pseudo-differentiation” [27]. Accordingly, few studies showed that high CK20 expression rates are associated with poor survival outcome in NMIBCs [26,71], in keeping with the findings from the gene-expression profiling study by Eckstein et al. on a cohort of MIBCs [127]. Nevertheless, results might be biased by the fact that a limited panel of markers under-estimated the high heterogeneity of the luminal class of BCs, which encompasses tumors with different molecular features and inherent outcomes.

### 3.6. FOXA1

*FOXA1*, a transcription factor involved in the binding of other transcription factors on chromatin, is already regarded as marker of luminal A breast tumors [129]. Previous studies based on preclinical models suggested that *FOXA1* may directly contribute to the establishment of the luminal subtype, and that its cooperation with *GATA3* and *PPARγ* is capable to reprogram basal cells to a luminal molecular subtype [130,131,132].

Though less used as a luminal marker of BC as compared to CK20 and GATA3, available data suggest its association with less aggressive NMIBCs and MIBCs, in keeping with the luminal subtype [101,133].

### 3.7. GATA3

GATA3 (binding protein) is a transcription factor routinely used as a urothelial and breast lineage-restricted marker, due to its involvement in regulating the luminal differentiation in these two types of epithelia [53,107], and it is also expressed in a subset of T lymphocytes [134]. Accordingly, in the study by Serag Eldien et al., GATA3 was detected in 85% of the studied cases, which was within the range of the highly variable rates of GATA3 expression in UC (<5–100%) [33,58,69,85,116,135]. GATA3 is known to be significantly less expressed in tumors with squamous morphology [69,136].

GATA3 immunoreactivity has been shown to be inversely related to grade, stage, PNI, LVI, the presence of nodal metastases, and an overall poor clinical outcome [69,116,117,137], both as a direct consequence of down-regulation to loss of GATA3, resulting in neoplastic transformation, migration, and invasion of urothelial-derived tumor cells through up-regulation of oncogenes [138,139], and because of the association between GATA3 down-regulation and up-regulation of epithelial-mesenchymal transition (EMT) molecules, vascular endothelial growth factor (VEGF) and matrix metalloproteinases (MMP-2 and MMP-9) [140], the latter being consistently involved in cancer cell migration/invasion, angiogenesis, tumor progression, and metastasis [141].

There is also an inverse relation between GATA3 expression and tumor proliferation in terms of mitotic rate and Ki67 index [58,69], possibly due to impairment of G0/G1 cell cycle checkpoint, causing significant G2/M and S phases arrest [137]. 

GATA3 has been used as a marker of the luminal subtype by some authors [33], yet its diffuse expression across MIBCs might prevent its ability to accurately discriminate between luminal and basal tumors [142]. 

### 3.8. P53

*P53* mutations are the most common genetic alterations identified in MIBC [143]. The P53-like subtype, defined by the accumulation of *P53* gene mutations, has been associated with chemoresistance to cisplatin-based treatment regimens [23,108,144]. In a large meta-cohort analysis of 2411 BCs, SCC and the HER2-like subtypes were enriched with *TP53* mutations, the former carrying a poorer prognosis [50]. Recently, Bontoux et al. reported high levels of P53 expression in their “not classified” subgroup, encompassing those cases which did not fit the dichotomous basal/luminal classification according to the expression of a four-antibody panel, including CK5/6, CK14, GATA3, and FOXA1; hence, the authors hypothesized that these tumors might belong to the LumU group enlisted in the recent consensus molecular classification [25,101].

According to the consensus classification, *P53* mutation rates were as high as 76%, 61%, 45%, and 32% in the LumU, Ba/Sq, LumNS, and LumP, respectively [25]. In keeping with that, since assessment of P53 expression by IHC is currently used as a marker of its mutation status [56,145,146,147], Olkhov-Mitsel et al. recently reported that aberrant P53 expression, defined as lack or excessive (>50%) staining, was identified in 80.2%, 48.0%, and 38.1% of MIBCs classified as GU, Basal, and Uro, respectively [56]. 

In the large meta-analysis by Dadhania et al., mesenchymal immunohistochemical markers, including smooth muscle actin, myosin, calponin, and desmin, were used to identify tumors labeled as p53-like [33]. Nevertheless, such markers stained the stromal component only, with tumor cells being mostly negative, in keeping with the reverse phase protein data available from one of the study cohorts. Hence, it was suggested that the p53 subtype might result from stromal contaminations of smooth muscle and myofibroblastic cells, rather than being an intrinsic BC subtype. Such “infiltrated” subset of BCs, corresponding to the TCGA II class of tumors [29], is characterized by resistance to cisplatin-based chemotherapy [23], and sensitivity to immune checkpoint inhibitors (atezolizumab) in the metastatic/unresectable setting [24,49].

### 3.9. P63 and P40

Other commonly used basal markers are P63 and its isoform P40 (ΔNp63). *P63* is a transcription factor which, along with *P40*, may function as either an oncogene or a tumor suppressor gene, and is involved in the regulation of basal gene-expression signature and epithelial stratification [23,65]. In NMIBC, P40 is associated with high-grade and high-risk disease, showing worse recurrence and survival rates [148].

Basal-like tumors defined by their CK5/6 expression, are enriched for *P63*-associated genes [146]. P63 was consistently expressed in most UroA and UroB tumor cells, except for the most luminal cell layer, likewise the normal urothelium, and its expression rate was significantly higher as compared to SCCL and GU tumors [65].

In the study by Sikic et al., hierarchical clustering showed an association between P63 and luminal markers in their large cohort of UTUCs [66], whereas P63 usually controls MYC expression in human BC cells, the latter being a common marker of basal tumors [149].

## 4. Conclusions

Molecular subtyping of bladder cancer might be an effective tool in establishing patients’ outcomes and responsiveness to different treatments. Nevertheless, mRNA-based profiling of tumors is too complex, money- and time-consuming to be implemented in clinical practice, therefore, immunohistochemistry-based algorithms have been proposed. Current data suggest that selected antibody panels applied to FFPE specimens may have a prognostic and predictive role. Furthermore, it can be assumed that incorporating IHC-based models of molecular subtyping, along with clinical and pathological parameters into existing algorithms might increase their efficacy. In this setting, a deep knowledge of each marker’s biological features in the setting of BC carcinogenesis, as well as BC intra- and intertumoral heterogeneity, is needed in order to further validate the available findings in multicenter studies.

## Figures and Tables

**Table 1:** Summary of the main molecular classification systems in BC [21,22,23,24,25,26,27,28].

UNC *	MDA **	Lund ***	TCGA ****	Consensus Classification
Basal-like	Basal	SCC-like	Basal–squamous	Basal–Squamous
Luminal	Luminal	UroA	Luminal	Luminal–Papillary (LumP)
	p53-like	UroB	Luminal–papillary	Luminal Non-Specified (LumNS)
		Infiltrated	Luminal-infiltrated	Luminal Unstable (LumU)
		Genomically Unstable	Neuronal	Stroma-rich
				Neuroendocrine-like (NE-like)

* University of North Carolina; ** MD Anderson Cancer Center; *** University of Lund; and **** The Cancer Genome Atlas.

**Table 2:** Potential responsiveness to different treatments according to the Consensus Classification [25].

**Molecular subtypes** **according to the** **Consensus** **Classification**	LumP	LumNS	LumU	Stroma-rich	Ba/Sq	NE-like
**Potential** **responsiveness** **to treatment**	FGFR3- targeted therapies	NAC, immunotherapy	Radiotherapy, immunotherapy	-	EGFR-targeted therapies, immunotherapy, NAC	Radiotherapy, immunotherapy

**Table 3:** Antibodies and clones used in selected studies (see text).

	Basal Markers										Luminal Markers						
	CD44		CK5 or CK5/6		CK14		P40		P63		CK20		FOXA1		GATA3		
	Antibody	Clone	Antibody	Clone	Antibody	Clone	Antibody	Clone	Antibody	Clone	Antibody	Clone	Antibody	Clone	Antibody	Clone	Ref.
NMIBC			+	XM26											+	L50-823	[78]
NMIBC, MIBC			+	D5/16 B4	+	LL002							+	Q-6	+	L50-823	[100]
MIBC	+	DF1485	+	XM26	+	SP53					+	Ks20.8	+	ab23738	+	L50-823	[44]
NMIBC (CIS)			+	D5/16 B4							+	Ks20.8					[88]
MIBC			+	D5/16 B4	+	LL002					+	Ks20.8			+	L50-823	[107]
MIBC			+	D5/16 B4							+	Ks20.8					[108]
NMIBC	+	DF1485	+	D5/16	+	OIT4A7					+	OTI4A2			+	UMAB218	[81]
NMIBC			+	D5/16 B4			+	BC28	+	4A4	+	SP33			+	L50-823	[83]
NMIBC, MIBC			+	D5/16 B4											+	L50-823	[68]
NMIBC, MIBC			+	D5/16	+	OIT4A7					+	OTI4A			+	UMAB218	[67]
MIBC			+	D5/16 B4	+	LL002					+	Ks20.8			+	HG3-31	[27]
